# Secondary structure spatial conformation footprint: a novel method for fast protein structure comparison and classification

**DOI:** 10.1186/1472-6807-6-12

**Published:** 2006-06-08

**Authors:** Elena Zotenko, Dianne P O'Leary, Teresa M Przytycka

**Affiliations:** 1 Department of Computer Science, University of Maryland,College Park, MD 20742, USA; 2 Institute for Advanced Computer Studies, University of Maryland, College Park, MD 20742,USA; 3 National Center for Biotechnology Information, National Library of Medicine, National Institutes of Health, Bethesda, MD 20894, USA

## Abstract

**Background:**

Recently a new class of methods for fast protein structure comparison has emerged. We call the methods in this class *projection methods *as they rely on a mapping of protein structure into a high-dimensional vector space. Once the mapping is done, the structure comparison is reduced to distance computation between corresponding vectors. As structural similarity is approximated by distance between projections, the success of any projection method depends on how well its mapping function is able to capture the salient features of protein structure. There is no agreement on what constitutes a good projection technique and the three currently known projection methods utilize very different approaches to the mapping construction, both in terms of what structural elements are included and how this information is integrated to produce a vector representation.

**Results:**

In this paper we propose a novel projection method that uses secondary structure information to produce the mapping. First, a diverse set of spatial arrangements of triplets of secondary structure elements, a set of *structural models*, is automatically selected. Then, each protein structure is mapped into a high-dimensional vector of "counts" or *footprint*, where each count corresponds to the number of times a given structural model is observed in the structure, weighted by the precision with which the model is reproduced. We perform the first comprehensive evaluation of our method together with all other currently known projection methods.

**Conclusion:**

The results of our evaluation suggest that the type of structural information used by a projection method affects the ability of the method to detect structural similarity. In particular, our method that uses the spatial conformations of triplets of secondary structure elements outperforms other methods in most of the tests.

## Background

The extensive collection of protein sequence and structure information has resulted in the creation of numerous classification resources for organizing proteins [1]. Two main structure-based classification databases, SCOP [2] and CATH [3], combine sequence, structural, and functional information to provide a hierarchical classification of known protein structures in the Protein Data Bank (PDB) [4]. In the SCOP database, for example, proteins are organized into a four-level hierarchy: class, fold, super-family, and family. Members of the same family group share a clear common evolutionary origin, supported either by significant sequence similarity or significant structural and functional similarity. The families are grouped into super-families based on structural or functional similarity that suggest a probable common evolutionary origin. The fold level groups proteins based on the arrangement of major secondary structure elements. And finally the class level groups proteins according to their secondary structure element content: mainly *α*, mainly *β*, mixed *α *and *β*, or small structures.

Classification hierarchy levels that group evolutionarily related or structurally similar proteins provide important insight into the evolution and functional relation between proteins; therefore the development of fast automatic methods for reliable relationship detection at these levels is an active area of research. Due to the absence of significant sequence similarity, relationships between distant *homologs *(evolutionarily related proteins) and *analogs *(structurally similar but evolutionarily unrelated proteins) are the most difficult to detect. Despite recent advances in sequence-based approaches, these relationships can still only be detected by protein structure comparison methods.

**Figure 1:**
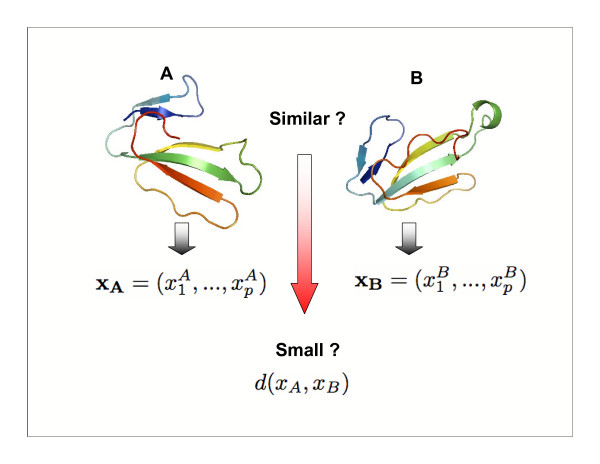
**Protein structure comparison via projection**. To compare structures *A *and *B*, a projection method will first map them to a vector in a high-dimensional vector space. Thus, structure *A *is mapped to vector ***x***_***A ***_and structure B to ***x***_***B***_. The structure comparison is then reduced to the distance computation between these vectors, i.e., the structures are similar if distance *d*(***x***_***A***_, ***x***_***B***_) is small.

In the past few decades numerous protein structure comparison methods have been proposed [5-10]. Because of the inherent difficulty of structural alignment, accurate residue-based alignment methods remain computationally expensive. To allow high-throughput protein structure comparison and classification, a number of less accurate but very fast protein structure comparison methods have been developed [8,11-21]. These methods fall into two basic categories. One group of methods [8,11-13,15,18,21] first identifies potentially equivalent structural elements and then finds a maximal consistent set of such elements using either dynamic programming or graph theoretical methods. A second, more recently pursued, class of methods [14,17,19,20] first maps a protein structure into a high-dimensional vector space. Once the mapping is done, the structure comparison is reduced to a distance computation between corresponding vectors, as shown in Figure 1, and therefore is extremely fast and simple. We refer to this class of methods as *projection methods*.

Even though projection methods do not produce a structural alignment as the result of comparison, there are two key applications in high-throughput comparative structure analysis, screening and classification, that may benefit from the ability of projection methods to perform fast and simple protein structure comparison. Protein structure alignment servers are routinely used to compare a query protein structure against a large database of structures such as the PDB. In the screening application, a projection method can be used to rank the structures in the database, allowing the more computationally expensive residue-based structure alignment method to be applied only to the highest ranked (small) fraction of the database. In the classification application a query structure has to be assigned to one of the groups of structures in the database (for example, we may want to assign a newly discovered structure to the correct super-family group in the SCOP classification database). Furthermore, a vector representation of protein structure produced by a projection approach can be combined with machine learning techniques to provide powerful classification schemes. Therefore improving the performance of projection methods and understanding the limits of these techniques is particularly important.

The central question in projection methods' approach to protein structure comparison is how to devise a mapping that is able to capture all the salient features of protein structure. Over the past few years three projection methods have been proposed that employ very different approaches to the mapping construction. In PRIDE2 [19,20], Gaspari *et al*. compute all pairwise distances between the central carbon atoms *k *residues apart (*k *ranging between three and thirty), and use the distance distributions as a descriptor of protein structure. In SGM [14], Rogen *et al*. map a polygonal line passing through the *C*_*α *_atoms of protein backbone into *R*^30 ^using geometric invariants borrowed from Knot Theory. In LFF [17], Choi *et al*. apply an idea common to diverse application areas, such as text mining [22] and classification of biological networks [23], in which a complex object is represented as a high-dimensional vector of counts or *footprint *of its small size motifs. In the case of protein structure, such motifs correspond to structural fragments. Choi *et al*. use pairs of backbone segments of size ten as structural fragments. Since the space of all such fragments is not discrete, a finite set of representative structural fragments or *models *is selected. Given a protein structure, its structural footprint is computed by making each structural fragment in the structure contribute a count of one to the closest (most similar) model. In this work we propose a novel projection method, *SSE Footprint (SSEF)*, that utilizes the structural footprinting paradigm and secondary structure information. Even though many protein structure comparison methods use secondary structure information to speed up the computation [8,11-13,15,16,21] (the list is by no means exhaustive) and some of them use pairs (or triplets) of secondary structure elements [8,11,15,16], we are not aware of any method that uses triplets of secondary structure elements as structural fragments to produce a vector representation of the protein structure as a whole. We argue in the next paragraph that triplets of secondary structures are particularly suitable for producing such a representation.

As projection methods approximate structural similarity by distance between corresponding vectors, the success of such methods depends critically on the choice of the mapping function. In particular, the mapping function should be able to tolerate a certain amount of variability in the less conserved regions of distantly related structures. It has been established that secondary structure elements are more conserved than loop regions, regions of the backbone in between the secondary structure elements. Therefore we reasoned that the mapping best suited for our purpose should capture the arrangement of secondary structure elements. Towards this end we chose a set of models that represents a large variety of possible conformations of triplets of secondary structure elements. Furthermore, we note that if, as in the LFF method, each structural fragment contributes only to the closest model, then the footprint is indeed a vector of counts with each dimension being the number of appearances of the corresponding model in the structure. Although this approach has an intuitive interpretation, it may be unstable when a structural fragment is almost equidistant from several models. To solve this problem, we allow a structural fragment to contribute to several models, with the most similar models getting the biggest contribution.

To evaluate our projection approach we perform a comprehensive comparison of our method with all currently known projection methods: LFF [17], SGM [14], and PRIDE2 [19,20]. The objective of our evaluation is to find out how well the methods perform in the context of two proposed application areas, screening and classification, and to understand what structural information is important for good performance. The later is possible as the methods evaluated use very different approaches to project a protein structure into a high-dimensional vector space. Finally we measure the running time of the methods on a massive all-against-all structure comparison. We conclude the paper by discussing a potential connection between the type of structural information captured by the mapping and the performance of each projection method.

## Results and discussion

### SSE footprint method

There are three main components in the general framework that underpins structural footprinting: selecting a type of structural fragment, selecting a representative set of structural fragments as models, and computing the footprint. The type of structural fragment that is chosen to model protein structure and its representation may affect greatly the ability of the mapping to be effective in detecting pairs of similar structures, especially distantly related structures. In our method we use a triplet of secondary structure elements as a structural fragment. We approximate each secondary structure by a positional vector in 3D (an SSE vector) and represent the spatial conformation of an SSE triplet by a robust descriptor that captures the relative orientation of the corresponding SSE vectors.

**Figure 2:**
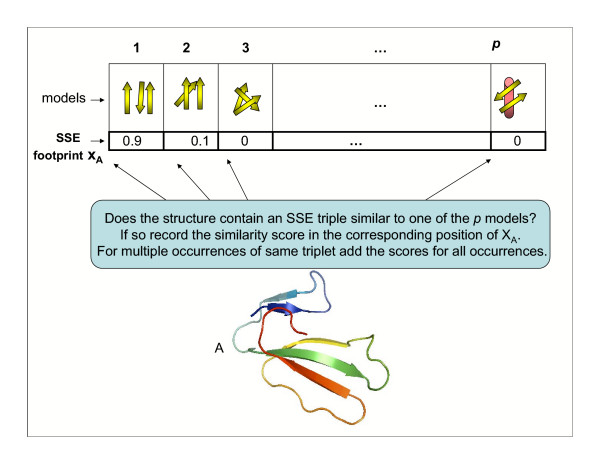
**Computing an SSE footprint**. Once a set of *p *models is selected, each protein domain is mapped to a vector in *R*^*p*^, where each dimension corresponds to a particular model and records the "weighted" number of times the model is observed in the structure of the domain. Here a protein structure A is mapped to an SSE footprint ***x***_***A***_.

We select a set of *p *spatial conformations as models via a clustering technique applied to SSE triplets extracted from a representative set of protein structures, in our case a set of fold representatives from every fold in the SCOP 1.65 classification database. Once the models are selected, each protein domain is mapped to a vector in *R*^*p*^, where each dimension corresponds to a particular model and records the "weighted" number of times the model is observed in the structure of the domain (see Figure 2 and Methods).

### Comprehensive evaluation of projection methods

For the results shown below we used the SCOP classification database version 1.65 (released on August, 2003). We repeated all the experiments with the latest versions of the SCOP version 1.69 [see Additional file 2, Additional file 3, Additional file 4, and Additional file 5] and the CATH version 2.6 [see Additional file 6, Additional file 7, and Additional file 8] databases. In all performance evaluation tests we took a set of non-redundant proteins extracted either at 40% sequence identity (for tests done with the SCOP database) or at 35% sequence identity (for tests done with the CATH database) as a set of database proteins.

#### Performance in the screening application

In the screening application the set of similar protein domains depends not only on the query and the database, but also to some extent on the protein structure alignment method that is being sped up. To decouple our evaluation procedure from a particular protein structure alignment method and to evaluate the method's performance as a stand-alone application, we turn to the gold standard, the SCOP classification database [2], for the definition of structurally similar protein domains or *true relationships *(The performance of all the methods with respect to the CATH classification database is similar). In this work we use three SCOP classification levels to define true relationships. To measure a method's ability to detect structural similarity between closely related domains we use SCOP family level, i.e., we say that a pair of domains is related if they belong to the same SCOP family group and unrelated otherwise. To measure the method's ability to detect structural similarity between distantly related protein domains, on the other hand, we use SCOP super-family and fold levels.

To measure how well a projection method performs in the screening application we use a variation of widely used ROC(*n*) curves. Given a projection method and a database of protein domains, each query protein domain defines a curve, which plots *coverage *(the fraction of related protein domains retrieved) against the *number of errors *(the number of unrelated domains retrieved). To obtain one curve per projection method that shows the method's performance for queries from different classification groups, the individual curves were averaged, first across different queries in the same classification group and then across different classification groups (see Methods).

**Figure 3:**
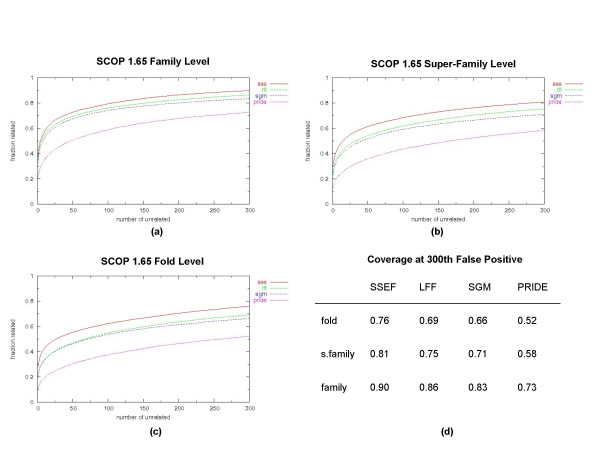
**Coverage versus error plots**. Coverage versus Error plots for the SSEF, LFF, SGM, and PRIDE2 methods. Given a projection method and a database of protein domains, each query protein domain defines a curve, which plots *coverage *(the fraction of related protein domains retrieved) against the *number of errors *(the number of unrelated domains retrieved). To obtain one curve per projection method the individual curves were averaged, first across different queries in the same classification group and then across different classification groups (see Methods). (a) Pairs in the same SCOP family are true positives; pairs in different SCOP families are false positives. (b) Pairs in the same SCOP super-family are true positives; pairs in different SCOP super-families are false positives. (c) Pairs in the same SCOP fold are true positives; pairs in different SCOP folds are false positives. (d) Coverage obtained by projection methods at different classification levels when 300th false positive result is encountered.

The Coverage versus Error plots for SCOP family, super-family and fold classification levels are shown in Figure 3(a)–(c). Even though each false positive result is an overhead for the structural alignment method being sped up with the screening, it is reasonable to assume that a few such errors can be tolerated as long as most of the related (similar) domains are retrieved. Thus, it is interesting to compare the coverage of different projection methods when the *n*th error is encountered. Here we show the coverage up to the 300th error, where 300 is about 5% (the actual number depends on the query) of the total number of unrelated domains in the database; the coverage for different methods when the 300th error is encountered is given in Figure 3(d). For example at the SCOP super-family level, the SSEF, LFF, SGM, and PRIDE methods retrieve 80.79%, 75.30%, 71.02%, and 58.50% of related domains respectively.

As expected, structural similarity between distantly related domains is more difficult to detect than structural similarity between close homologs for all four methods. Thus, at the SCOP family level, the three best methods achieve 83% – 90% coverage at the 300th false positive and only 66% – 76% at the SCOP fold level. While the SSEF method has better performance at all classification levels, the difference is most profound at the SCOP super-family and fold levels. The SGM and LFF methods have comparable performance at the lower error levels, but at the higher error levels the LFF gains about 4% in coverage over the SGM. The PRIDE2 method has worse performance than the other three methods.

#### Performance in the classification application

To evaluate the performance in the classification application, we reproduce the SCOP classification hierarchy using a nearest neighbor classification strategy. This classification scheme assigns a protein domain to the group of its nearest neighbor in the database (see Methods). We compute two numbers per projection method and per classification level in the SCOP database. The first number ("% accuracy") is the percentage of domains that are classified correctly, i.e., the fraction of domains whose nearest neighbor belongs to the same classification group as the domain itself. The second number ("% accuracy over groups") is an attempt to remove the bias towards large classification groups. To obtain this number we first compute the fraction of correctly classified domains within each group and then average across the groups.

The classification accuracies for the methods are given in Table 1. Comparison of "% accuracy" and "% accuracy over groups" numbers shows that the nearest neighbor classification strategy is biased towards large groups. But the difference in performance between methods is consistent; the SSEF method has the best accuracy at the super-family and fold levels while the LFF method has the best accuracy at the family level.

The best classification accuracies ("% accuracy over groups"), 58.3% at the family level (the LFF method), 58.8% at the super-family level (the SSEF method), and 62.8% at the fold level (the SSEF method), indicate that there is room for improvement. Manual inspection of accuracies of different groups revealed that some groups are hard to classify for some projection methods and easy for others. For example, the LFF method classifies both members of the g. 4.1 group (*PMP inhibitors*) while the SSEF has 0% accuracy for this group. The roles are reversed for the a. 60. 6 group (*DNA polymerase beta, N-terminal domain-like*); the SSEF method classifies both members correctly and the LFF none.

#### Running time

To compare the efficiency of the projection methods evaluated in this study we analyze for each method the running time needed to performall-against-all structure comparison of 5,345 domains in the SCOP 40%-id dataset. All programs were run on a Linux machine with an Intel Xeon CPU 3.20 GHz and the results are shown in Table 2.

For any projection method the all-against-all structure comparison involves two steps: the first step is the pre-processing step where the structures are projected into vectors, and the second step is the pairwise distance computation between the set of vectors. If there are *n *structures in the dataset then the total running time is *n *× *prep *+ n(n−1)2
 MathType@MTEF@5@5@+=feaafiart1ev1aaatCvAUfKttLearuWrP9MDH5MBPbIqV92AaeXatLxBI9gBaebbnrfifHhDYfgasaacH8akY=wiFfYdH8Gipec8Eeeu0xXdbba9frFj0=OqFfea0dXdd9vqai=hGuQ8kuc9pgc9s8qqaq=dirpe0xb9q8qiLsFr0=vr0=vr0dc8meaabaqaciaacaGaaeqabaqabeGadaaakeaadaWcaaqaaiabd6gaUjabcIcaOiabd6gaUjabgkHiTiabigdaXiabcMcaPaqaaiabikdaYaaaaaa@3407@ × *eval*, where *prep *is the average pre-processing time per structure and *eval *is the average time to compare a pair of structures. It should be noted that we use the pre-processing to denote the mapping of each structure into a vector, i.e., no pairwise computations are done during this step.

For applications of screening and classifications, we can assume that the pre-processing step is done once for the database proteins and therefore the running time spent on in this step is amortized as the number of queries against the database grows. Even though the pre-processing step does not affect directly the effectiveness of a projection method, it may be indicative of the "amount of information" that is used during projection computation. For example, the LFF method computes a very detailed description of the structural fragments it uses to generate the mapping; each pair of backbone segments of length ten is described by 10 × 10 inter-atomic distance matrix between the *C*_*α *_atoms.

The running time spent on distance computations is mainly affected by the dimension of the projection, *p*. Our method uses *p *= 1,500 and takes about 10 times longer to compute the distances than the LFF (*p *= 100) and SGM (*p *= 30) methods. But even the 1,054 seconds to perform 5,345 * (5, 345 - 1)/2 = 14,281, 840 protein structure comparisons is almost negligible compared to the time it would take DALI [7] to perform the same number of comparisons. We have used the DaliLite program [24] and estimated that one query against the same database of 5, 345 domains takes on average 4,800 seconds or 1.3 hours. Therefore, unless a screening method is applied,the entire all-against-all comparison would take about 3,474 hours or 4.825 months to compute.

## Conclusion

In this work we described a novel projection method for protein structure comparison. Our method is different from other projection methods in that it uses the relative orientation of triplets of secondary structure elements in the projection computation. An extensive comparison to other currently known projection methods indicates that the projection technique used by our method better captures features of protein structure important for detecting structural similarity at all levels: from the structural similarity characteristic of closely related structures to the structural similarity characteristic of distantly related protein domains. Moreover, the performance of our method is stable with respect to the secondary structure assignment algorithm used to define the SSEs. In the early stages of our work we used the secondary structure assignment from the MMDB database [25] and found that the performance of our method does not depend on the actual secondary structure assignment, i.e., both the MMDB and the DSSP (used now) assignment schemes result in very similar performance.

Our evaluation procedure concentrated on performance in two application areas uniquely suited for projection methods: screening and classification. The difference in performance between the methods is consistent across application areas and also across different classification databases (results for the SCOP version 1.69 and the CATH version 2.6 are supplied as supplementary material), which allows us to speculate about the appropriateness of different projection techniques for protein structure comparison. Based on our evaluation it seems that interaction patterns between atoms at most thirty residues apart do not carry a strong enough signal and the projection technique that uses this structural information alone did not perform well in our tests. We have been experimenting with the geometric invariants used by the SGM method, and they appear to emphasize local spatial interactions between line segments connecting neighboring *C*_*α *_atoms. As the LFF method puts a 20°*A *threshold on the maximum distance between *C*_*α *_atoms, both the LFF and the SGM methods capture local spatial interactions between residues and good (and also comparable) performance of both methods suggests these interactions are enough to capture structural similarity. Finally, as indicated by the performance of SSEF method, information about spatial conformation of triplets of secondary structure elements gives an edge, especially in detecting structural similarity between distantly related domains.

As projection methods produce a representation of protein structure in a vector form, they open the door to the application of machine learning techniques, such as support vector machines, to the task of protein structure classification. In this work we have used a simple classification scheme, the nearest neighbor classification, in the classification experiments. In our future work we plan to combine the SSEF method with more powerful classification strategies to improve the classification accuracy. Another direction for improvement stems from the fact that difficult-to-classify groups are not uniform across the methods. Therefore, we also plan to investigate how classification decisions produced from different methods can be combined to obtain better classification accuracy.

## Methods

### SSE footprint method

#### SSE triplets and their representation

We use a triplet of secondary structure elements (SSEs) as a structural fragment. The secondary structure assignment is computed by the DSSP program [26] and each secondary structure element is approximated by a positional vector in 3D or *an SSE vector*. The SSEs are either *α*-helices or *β*-strands, so in addition to the positional information given by a triplet of vectors in 3D, each structural fragment is assigned a type: *α α α, α α β,α β α,α β β, β α α, β α β, β β α *or *β β β*, according to the type of secondary structure elements that it contains.

Since the relative orientation of distant pairs of secondary structure elements is less stable, we restrict our consideration to triplets that are close in space, requiring each of the three pairwise distances between the midpoints of SSE vectors to be less than a certain threshold. The adoption of "local" SSE triplets as a structural fragment also reduces the effect of an occasional SSE insertion/deletion on footprints of related domains. For example, consider a pair of related domains, one having *n *SSEs and the other having *n *+ 1 SSEs. Without any restrictions the additional SSE may generate up to *n*^2 ^SSE triplets that will register in the footprint of one structure but not the other. By considering only local SSE triplets the impact of such insertions/deletions is considerably reduced. The particular value of 30*Å *that we have adopted reflects a trade-off between noise and the ability to map every structure to an SSE footprint. Smaller threshold values result in a large number of structures with three or more SSEs but no valid SSE triplets. Larger threshold values result in a worse performance as the spatial orientation of triplets becomes less stable and the effect of SSE insertion/deletion grows.

The spatial conformation of an SSE triplet is represented by all pairwise angles and all pairwise distances between the midpoints of the corresponding SSE vectors. Since angles and distances are measured in different units, a standard normalization procedure is applied, normalizing a quantity *x *by x−meanxstdevx
 MathType@MTEF@5@5@+=feaafiart1ev1aaatCvAUfKttLearuWrP9MDH5MBPbIqV92AaeXatLxBI9gBaebbnrfifHhDYfgasaacH8akY=wiFfYdH8Gipec8Eeeu0xXdbba9frFj0=OqFfea0dXdd9vqai=hGuQ8kuc9pgc9s8qqaq=dirpe0xb9q8qiLsFr0=vr0=vr0dc8meaabaqaciaacaGaaeqabaqabeGadaaakeaadaWcaaqaaiabdIha4jabgkHiTiabb2gaTjabbwgaLjabbggaHjabb6gaUnaaBaaaleaacqWG4baEaeqaaaGcbaGaee4CamNaeeiDaqNaeeizaqMaeeyzauMaeeODay3aaSbaaSqaaiabdIha4bqabaaaaaaa@3EC3@. The mean and the standard deviation are computed from the distribution of angle and distance values in triplets of the SSE vectors corresponding to structural fragments extracted from the SCOP fold dataset. Given a pair of structural fragments, their distance is then measured by the Euclidean norm of the difference between corresponding vectors. From the point of view of protein structure a triplet of *α*-helices is quite different from a triplet of *β*-strands even if their spatial conformation is similar, therefore in our algorithm we never mix SSE triplets of different types and treat them separately as explained later on.

#### Selection of models

To obtain a representative set of fragments, we first extract all triplets of secondary structure elements from protein domains in the SCOP fold dataset. The triplets are then divided into eight groups based on their type, and each group is clustered with a *k*-means clustering algorithm to obtain a total of *p *clusters. The cluster centers are chosen as the models. Moreover, the models acquire the type of the group that they come from.

Since triplets of secondary structure elements with a majority of *β*-strands are more abundant than other triplets, the *ααα, ααβ, αβα*, and *βαα *groups contain fewer structural fragments than the *αββ, βαβ, ββα *and *βββ *groups. We compensate for such an uneven distribution by allocating 1.5 more clusters to groups with a majority of *β*-strands. Thus to get a total of *p *= 1,500 models, we allocate 225 clusters each to *αββ, βαβ, ββα *and *βββ *groups and 150 clusters each to *ααα, ααβ, αβα*, and *βαα *groups.

#### Footprint computation

The footprint of a structure *Q *is a vector in *R*^*p*^, where each dimension corresponds to a specific model and its value is equal to a "count" accumulated by the model over all structural fragments in *Q*. As mentioned before, to achieve stability of the method, we allow a structural fragment to contribute to several models, where the amount of contribution is inversely proportional to the distance between the fragment and the model. A footprint is formally defined as follows.

fQ     (f1Q,…,fpQ)fiQ     ∑sc(s,mi)c(s,mi)     exp⁡(−d(s,mi)2/a)∑mjexp⁡(−d(s,mj)2/a)     is a structural fragment of Qc(s,mi)     is a contribution of s to model mid(s,mi)     is the distance between s and a model mi     is a scale factor
 MathType@MTEF@5@5@+=feaafiart1ev1aaatCvAUfKttLearuWrP9MDH5MBPbIqV92AaeXatLxBI9gBaebbnrfifHhDYfgasaacH8akY=wiFfYdH8Gipec8Eeeu0xXdbba9frFj0=OqFfea0dXdd9vqai=hGuQ8kuc9pgc9s8qqaq=dirpe0xb9q8qiLsFr0=vr0=vr0dc8meaabaqaciaacaGaaeqabaqabeGadaaakqGabeWacWcbbGBbaGQbbaGaaCzcaGqadiab=zgaMnaaBaaaleaacqWFrbquaeqaaOGaaCzcaGqaciab+1da9iaaxMaadaqadaqaaiabdAgaMnaaDaaaleaacqaIXaqmaeaacqWGrbquaaGccqGGSaalcqWIMaYscqGGSaalcqWGMbGzdaqhaaWcbaGaemiCaahabaGaemyuaefaaaGccaGLOaGaayzkaaaabaGaaCzcaiabdAgaMnaaDaaaleaacqWGPbqAaeaacqWGrbquaaGccaWLjaGaeyypa0JaaCzcamaaqafabaGaem4yamgaleaacqWGZbWCaeqaniabggHiLdGccqGGOaakcqWGZbWCcqGGSaalcqWGTbqBdaWgaaWcbaGaemyAaKgabeaakiabcMcaPaqaaiaaxMaacqWGJbWycqGGOaakcqWGZbWCcqGGSaalcqWGTbqBdaWgaaWcbaGaemyAaKgabeaakiabcMcaPiaaxMaacqGH9aqpcaWLjaWaaSaaaeaacyGGLbqzcqGG4baEcqGGWbaCcqGGOaakcqGHsislcqWGKbazcqGGOaakcqWGZbWCcqGGSaalcqWGTbqBdaWgaaWcbaGaemyAaKgabeaakiabcMcaPmaaCaaaleqabaGaeGOmaidaaOGaei4la8IaemyyaeMaeiykaKcabaWaaabeaeaacyGGLbqzcqGG4baEcqGGWbaCcqGGOaakcqGHsislcqWGKbazcqGGOaakcqWGZbWCcqGGSaalcqWGTbqBdaWgaaWcbaGaemOAaOgabeaakiabcMcaPmaaCaaaleqabaGaeGOmaidaaaqaaiabd2gaTnaaBaaameaacqWGQbGAaeqaaaWcbeqdcqGHris5aOGaei4la8IaemyyaeMaeiykaKcaaaqaaiaaxMaacqWGZbWCcaWLjaGaaGPaVlaaxMaacqqGPbqAcqqGZbWCcqqGGaaicqqGHbqycqqGGaaicqqGZbWCcqqG0baDcqqGYbGCcqqG1bqDcqqGJbWycqqG0baDcqqG1bqDcqqGYbGCcqqGHbqycqqGSbaBcqqGGaaicqqGMbGzcqqGYbGCcqqGHbqycqqGNbWzcqqGTbqBcqqGLbqzcqqGUbGBcqqG0baDcqqGGaaicqqGVbWBcqqGMbGzcqqGGaaicqWGrbquaeaacaWLjaGaem4yamMaeiikaGIaem4CamNaeiilaWIaemyBa02aaSbaaSqaaiabdMgaPbqabaGccqGGPaqkcaWLjaGaaGPaVlaaxMaacqqGPbqAcqqGZbWCcqqGGaaicqqGHbqycqqGGaaicqqGJbWycqqGVbWBcqqGUbGBcqqG0baDcqqGYbGCcqqGPbqAcqqGIbGycqqG1bqDcqqG0baDcqqGPbqAcqqGVbWBcqqGUbGBcqqGGaaicqqGVbWBcqqGMbGzcqqGGaaicqWGZbWCcqqGGaaicqqG0baDcqqGVbWBcqqGGaaicqqGTbqBcqqGVbWBcqqGKbazcqqGLbqzcqqGSbaBcqqGGaaicqWGTbqBdaWgaaWcbaGaemyAaKgabeaaaOqaaiaaxMaacqWGKbazcqGGOaakcqWGZbWCcqGGSaalcqWGTbqBdaWgaaWcbaGaemyAaKgabeaakiabcMcaPiaaxMaacaaMc8UaaCzcaiabbMgaPjabbohaZjabbccaGiabbsha0jabbIgaOjabbwgaLjabbccaGiabbsgaKjabbMgaPjabbohaZjabbsha0jabbggaHjabb6gaUjabbogaJjabbwgaLjabbccaGiabbkgaIjabbwgaLjabbsha0jabbEha3jabbwgaLjabbwgaLjabb6gaUjabbccaGiabdohaZjabbccaGiabbggaHjabb6gaUjabbsgaKjabbccaGiabbggaHjabbccaGiabb2gaTjabb+gaVjabbsgaKjabbwgaLjabbYgaSjabbccaGiabd2gaTnaaBaaaleaacqWGPbqAaeqaaaGcbaGaaCzcaiabdggaHjaaxMaacaaMc8UaaCzcaiabbMgaPjabbohaZjabbccaGiabbggaHjabbccaGiabbohaZjabbogaJjabbggaHjabbYgaSjabbwgaLjabbccaGiabbAgaMjabbggaHjabbogaJjabbsha0jabb+gaVjabbkhaYbaaaa@3F9C@

We should note that due to the type separation mentioned above, a structural fragment contributes only to models of the same type, i.e., *c*(*s, m*_*i*_)*= *0 if *s *and *m*_*i *_are of different types. Moreover contributions of *s *to different models are normalized, so that the overall contribution of *s *sums up to one, i.e., ∑mic(s,mi)=1
 MathType@MTEF@5@5@+=feaafiart1ev1aaatCvAUfKttLearuWrP9MDH5MBPbIqV92AaeXatLxBI9gBaebbnrfifHhDYfgasaacH8akY=wiFfYdH8Gipec8Eeeu0xXdbba9frFj0=OqFfea0dXdd9vqai=hGuQ8kuc9pgc9s8qqaq=dirpe0xb9q8qiLsFr0=vr0=vr0dc8meaabaqaciaacaGaaeqabaqabeGadaaakeaadaaeqaqaaiabdogaJjabcIcaOiabdohaZjabcYcaSiabd2gaTnaaBaaaleaacqWGPbqAaeqaaOGaeiykaKIaeyypa0JaeGymaedaleaacqWGTbqBdaWgaaadbaGaemyAaKgabeaaaSqab0GaeyyeIuoaaaa@3BBF@. Once footprints are computed, a distance between two protein domains is measured by the Pearson correlation coefficient of their footprints ***f***_***Q ***_and ***f***_***P***_:

∑i=1p(fiQ−μQ)(fiP−μP)∑i=1p(fiQ−μQ)2∑i=1p(fiP−μP)2
 MathType@MTEF@5@5@+=feaafiart1ev1aaatCvAUfKttLearuWrP9MDH5MBPbIqV92AaeXatLxBI9gBaebbnrfifHhDYfgasaacH8akY=wiFfYdH8Gipec8Eeeu0xXdbba9frFj0=OqFfea0dXdd9vqai=hGuQ8kuc9pgc9s8qqaq=dirpe0xb9q8qiLsFr0=vr0=vr0dc8meaabaqaciaacaGaaeqabaqabeGadaaakeaadaWcaaqaamaaqadabaWaaeWaaeaacqWGMbGzdaqhaaWcbaGaemyAaKgabaGaemyuaefaaOGaeyOeI0ccciGae8hVd02aaSbaaSqaaiabdgfarbqabaaakiaawIcacaGLPaaadaqadaqaaiabdAgaMnaaDaaaleaacqWGPbqAaeaacqWGqbauaaGccqGHsislcqWF8oqBdaWgaaWcbaGaemiuaafabeaaaOGaayjkaiaawMcaaaWcbaGaemyAaKMaeyypa0JaeGymaedabaGaemiCaahaniabggHiLdaakeaadaGcaaqaamaaqadabaWaaeWaaeaacqWGMbGzdaqhaaWcbaGaemyAaKgabaGaemyuaefaaOGaeyOeI0Iae8hVd02aaSbaaSqaaiabdgfarbqabaaakiaawIcacaGLPaaadaahaaWcbeqaaiabikdaYaaaaeaacqWGPbqAcqGH9aqpcqaIXaqmaeaacqWGWbaCa0GaeyyeIuoaaSqabaGcdaGcaaqaamaaqadabaWaaeWaaeaacqWGMbGzdaqhaaWcbaGaemyAaKgabaGaemiuaafaaOGaeyOeI0Iae8hVd02aaSbaaSqaaiabdcfaqbqabaaakiaawIcacaGLPaaadaahaaWcbeqaaiabikdaYaaaaeaacqWGPbqAcqGH9aqpcqaIXaqmaeaacqWGWbaCa0GaeyyeIuoaaSqabaaaaaaa@69CE@

where *μ*_*Q *_and *μ*_*P *_are the means of ***f***_***Q ***_and ***f***_***P***_, respectively.

#### Selecting the number of models

**Figure 4:**
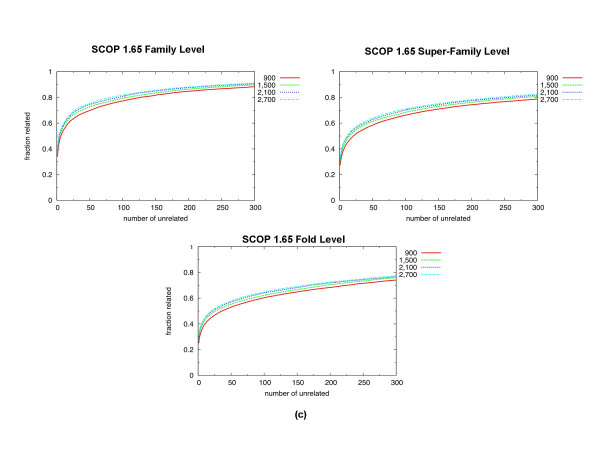
**Saturation of performance with the number of models**. Coverage versus Error plots for our method with different number of models: 900, 1, 500, 2,100 and 2, 700 models. (a) Pairs in the same SCOP family are true positives; pairs in different SCOP families are false positives. (b) Pairs in the same SCOP super-family are true positives; pairs in different SCOP super-families are false positives. (c) Pairs in the same SCOP fold are true positives; pairs in different SCOP folds are false positives.

One would expect the power of the mapping would increase with the number of models. To find the optimal number of models we have compared the performance of our method with 900, 1, 500, 2,100 and 2,700 models using Coverage versus Error plots described earlier (see Figure 4). While there is a clear difference in the performance between 900 and other configurations, the difference among 1, 500, 2,100 and 2,700 configurations is negligible. As the number of models determines the dimension of the projection and therefore affects the time spent on the distance computations, we decided to adopt *p *= 1,500. Another justification for using *p *= 1, 500 comes from an attempt to make the LFF and SSEF methods comparable. For the purpose of footprint computation, our method "sees" a structural fragment as a point in *R*^6^. By setting the number of models to *p *= 1,500, the total number of dimensions used is 1,500 × 6 = 9,000, which is comparable to the 100 × 100 = 10,000 used by LFF (100 models with a structural fragment being a point in *R*^100^).

### Data sets

For the results shown in the paper we used the SCOP classification database version 1.65 (released on August, 2003). We repeated all the experiments with the latest versions of SCOP version 1.69 (released on October, 2004) and CATH version 2.6 (released on April, 2005). For experiments based on the SCOP classification database, we downloaded a list of non-redundant domains filtered at 40% sequence identity (the SCOP 40%-id dataset) and the corresponding PDB-style files from the ASTRAL compendium [27]. For experiments based on the CATH classification database we downloaded a list of of non-redundant domains filtered at 35% sequence identity from the CATH classification database web-site. The PDB-style files were extracted based on the description of domains obtained from the CATH web-site.

As our method needs at least three secondary structure elements to compute the structural footprint, we excluded domains with fewer than three secondary structure elements, which resulted in datasets with 5,345 domains for SCOP version 1.65 [see Additional file 9], 6,902 domains for SCOP version 1.69 [see Additional file 10] and 5, 623 domains for CATH version 2.6 [see Additional file 11]. For model selection we used the SCOP fold dataset, a set of structures that represent every fold in the SCOP version 1.65 selected from the SCOP 40%-id dataset.

The SSEF and SGM methods were not able to produce a vector representation for some domains in the SCOP 40%-id (CATH 35%-id) datasets. Our method is not able to produce a vector representation when a protein domain does not have a single valid SSE triplet (see SSE triplets and their representation). For the SSEF the number of problematic domains was 11 out of 5, 345 (about 0.2%) domains for the SCOP version 1.65, 18 out of 6,902 (about 0.3%) for the SCOP version 1.69, and 30 out of 5, 623 (about 0.5%) domains for the CATH version 2.6. For the SGM the number of problematic domains was 419 out of 5,345 (about 7.0%) domains for the SCOP version 1.65, 601 out of 6,902 (about 8.7%) for the SCOP version 1.69, and 419 out of 5, 623 (about 7.8%) domains for the CATH version 2.6. Therefore the results reported for these methods are based on the datasets from which the problematic domains were removed.

### Computing coverage versus error plots

We first identify all protein domains that have at least four other related domains (domains that are in the same SCOP classification group) in the SCOP 40%-id dataset. We query all these domains against the SCOP 40%-id dataset and compute the individual Coverage versus Error curve for each query. The curve is computed by first ordering the protein domains in the SCOP 40%-id dataset by their structural similarity to the query domain. This ordered list is examined from the most similar to the least similar domain; for each false positive result (an unrelated domain) the fraction of related domains retrieved so far is recorded; the process stops when the 300th unrelated domain is encountered. The individual curves are then averaged, first across different queries in the same SCOP classification group and then across different SCOP classification groups.

### Computing nearest neighbor classification accuracies

To compute the numbers for a given projection method and classification level in Table 1 we first select a set of protein domains from the SCOP 40%-id dataset that have at least one additional domain in the same classification group. Then we compare each domain to the rest of the domains in the SCOP 40%-id dataset and record the classification group of its nearest neighbor. The "% accuracy" number is the fraction of domains correctly classified, i.e., whose nearest neighbor belongs to the same classification group as the domain itself. To obtain the "% accuracy over groups" number we first computed the fraction of correctly classified domains within each group and then averaged over the groups. The number of domains (classification groups) included in the computations for the SCOP 1.65 database are 5,058 (483), 4,826 (702), and 4,118 (989) for fold, super-family and family levels respectively.

### Programs

For our evaluations we obtained programs for the PRIDE2 and SGM methods from the authors of these methods. As the SGM program produced only the "raw" projections, we normalized the projections and computed distances as described in [14]. For the LFF method, we obtained the set of models from the authors of the LFF method. We computed footprints and distances as described in [17]. We obtained the DaliLite suite of programs [24] from the web-site of Liisa Holm's research group. We implemented the prototype of SSEF method in Python using the BioPython suite of packages [28]. The Python code and auxiliary files necessary to compute the SSE footprint from a PDB file of a structure are given as supplementary material [see Additional file 12].

## Authors' contributions

EZ carried out the experiments and drafted the manuscript. DPO helped to draft the manuscript and design the experiments. TMP conceived the project, supervised the project, and helped to draft the manuscript and design the experiments. All authors read and approved the final manuscript.

## Supplementary Material

Additional File 2 **SCOP 1.69 nearest neighbor classification**. An Excel workbook that contains nearest neighbor classification accuracies. Click here for file

Additional File 3 SCOP 1.69 coverage versus error for the fold level. Click here for file

Additional File 4 SCOP 1.69 coverage versus error for the super-family level. Click here for file

Additional File 5 SCOP 1.69 coverage versus error for the family level. Click here for file

Additional File 6 **CATH 2.6 nearest neighbor classification**. An Excel workbook that contains nearest neighbor classification accuracies. The PRIDE2 data is not shown due to technical difficulties of running the program on the CATH dataset. Click here for file

Additional File 7 **CATH 2.6 coverage versus error for the topology level**. The PRIDE2 data is not shown due to technical difficulties of running the program on the CATH dataset. Click here for file

Additional File 8 **CATH 2.6 coverage versus error for the homologous super-family level**. The PRIDE2 data is not shown due to technical difficulties of running the program on the CATH dataset. Click here for file

Additional File 1 **SCOP 1.65 nearest neighbor classification**. An Excel workbook that contains nearest neighbor classification accuracies. Click here for file

Additional File 9 SCOP 1.65 list of domains in the 40%-id dataset. Click here for file

Additional File 10 SCOP 1.69 list of domains in the 40%-id dataset. Click here for file

Additional File 11 CATH 2.6 list of domains in the 35%-id dataset. Click here for file

Additional File 12 **The Python code for the SSEF method**. An archive that contains the Python code and auxiliary files necessary to compute the SSE footprint from a PDB file of a structure. Click here for file

## Figures and Tables

**Table 1:** Classification accuracy. Agreement with the SCOP classification database. For every classification level the percentage of domains with a nearest neighbor in the same classification group is given for the SSEF, LFF, SGM, and PRIDE2 methods. The classification accuracy per group is given as supplementary material [see Additional file 1].

	% accuracy				% accuracy over groups			
SCOP	ssef	lff	sgm	pride	sse	lff	sgm	pride
fold	75.4	68.7	69.1	48.4	62.8	57.2	57.1	38.9
s.family	70.8	67.1	65.3	47.4	58.8	56.5	54.8	39.2
family	65.4	66.9	63.1	49.8	56.2	58.3	54.5	41.5

**Table 2:** Running time. The running time (in seconds) to perform all pairwise comparisons of 5,345 domains for the SSEF, LFF, SGM, and PRIDE2 methods. The running time is broken into running times spent on the pre-processing step and the distance computation step. The pre-processing step includes all the computation necessary to compute projections for 5, 345 domains. The distance computation step includes all pairwise distance computations between 5,345 projections computed in the pre-processing step. As the detailed information is not available for the PRIDE2 method, only the total time is shown for this method.

	Running time in seconds			
	ssef	lff	sgm	pride
pre-processing	3,067	490, 449	4,397	not available
distance computations	1,054	169	136	not available
total	4,121	490, 618	4,533	13, 200
